# An overview of psychological and social factors in Charles Bonnet syndrome

**DOI:** 10.1177/25158414211034715

**Published:** 2021-07-30

**Authors:** Lee Jones, Lara Ditzel-Finn, Jamie Enoch, Mariya Moosajee

**Affiliations:** NIHR Biomedical Research Centre, Moorfields Eye Hospital NHS Foundation Trust, London, UK; Institute of Ophthalmology, University College London, London, UK; Department of Ophthalmology, Great Ormond Street Hospital for Children NHS Trust, London, UK; Department of Optometry and Visual Sciences, City, University of London, London, UK; Institute of Ophthalmology, University College London, 11-43 Bath Street, London EC1V 9EL, UK; NIHR Biomedical Research Centre, Moorfields Eye Hospital NHS Foundation Trust, London, UK; Department of Ophthalmology, Great Ormond Street Hospital for Children NHS Trust, London, UK; The Francis Crick Institute, London, UK

**Keywords:** Charles Bonnet syndrome, patient experience, wellbeing

## Abstract

Charles Bonnet syndrome (CBS) is a condition where cognitively normal individuals with sight impairment experience simple and/or complex visual hallucinations. The exact pathogenesis of CBS is unknown; however, deafferentation is often recognised as a causal mechanism. Studies have provided insight into the multifaceted impact of CBS on wellbeing. Onset of CBS may cause distress among those believing visual hallucinations are indicative of a neurological condition. Hallucinatory content is often congruent with the emotional response. For example, hallucinations of a macabre nature typically result in a fearful response. Visual hallucinations may be highly disruptive, causing everyday tasks to become challenging. Clinical management relies on forewarning and pre-emptive questioning. Yet, knowledge and awareness of CBS is typically low. In this review, we provide a summary of the social and psychological implications of CBS and explore recent developments aimed at raising awareness and improving patient management.

## Introduction

Swiss neurologist Georges de Morsier is credited with coining the term Charles Bonnet syndrome (CBS) recognising the occurrence of visual hallucinations among individuals with impaired vision. Named after the 18th century philosopher, Charles Bonnet recounted his grandfather’s hallucinatory experiences in his published work *Essai analytique sur les facultés de l’âme* (Analytical Essay on the Faculties of the Soul; English translation). The condition is characterised by the presence of simple or complex visual hallucinations in patients with some form of sight impairment, occurring without conscious volition and with retained insight that what is observed during an episode is not real.^1^ Simple hallucinations usually consist of lights and flashes known as photopsia, whereas complex hallucinations involve formed images that can including faces, figures, animals and vivid scenery.^2^

CBS has been estimated to occur in 11–15% of those with sight loss.^2^ However, these estimates vary widely depending on the study, with more recent evidence indicating that as many as one in five low vision patients experience visual hallucinations.^3^ This variability reflects some the clinical challenges associated with CBS; for example, there has been a long-standing lack of consistency regarding diagnostic criteria.^1^ Visual hallucinations have a broad differential diagnosis, for instance, photopsia (presence of flashes or floaters) may precede other serious ocular conditions, such as retinal tears or detachment. Perceptual disturbances are usually characteristic of psychiatric disorders, and visual hallucinations may initially be investigated as psychotic or neurodegenerative conditions such as schizophrenia or dementia with Lewy Bodies. Accurate diagnosis of CBS relies on detailed elucidation of the clinical features and an understanding of alternative phenomena such as peduncular hallucinosis, a rare form of visual hallucinations often described in relation to both vascular and infective lesions of the mesencephalon and thalamus. There is also a degree of ambiguity surrounding associated risk factors of CBS onset. For example, old age and significantly reduced visual acuity are often regarded as contributing factors;^4,5^ however, a number of studies have observed visual hallucinations in the absence of these factors.^6–12^

**Figure 1.**
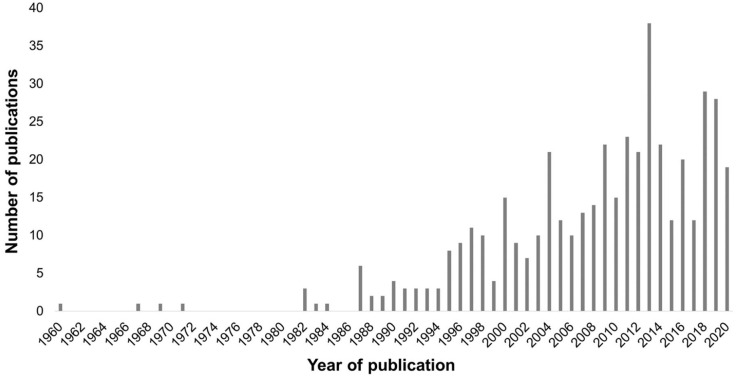
Number of publications by year in PubMed that include ‘Charles Bonnet syndrome’ in the article title or abstract (as of 11 March 2021).

Despite first being described in the 18th century, only quite recently has CBS attracted wider attention in the scientific community. For example, a search of the electronic research database PubMed reveals an upwards trend in publications relating to CBS, with almost 60% of the published papers emerging in the last 10 years (Figure 1). This increase in publications coincides with the adoption of CBS in the forthcoming version of the World Health Organization (WHO) International Classification of Diseases (ICD-11). Within the published literature, there is a particular focus on attempting to delineate the pathogenesis of CBS.^13^ Visual hallucinations are widely interpreted to be a consequence of deafferentation, whereby increased activity in the visual cortex occurs following reduced sensory input from the eyes.^14,15^ There have been fewer efforts to understand the psychological impact of CBS, which may partly be explained by the frequent assertion that many patients are not concerned about visual hallucinations or feel impartial or even relish their occurrence.^16^ Yet, those who suffer a negative response to CBS often do so acutely with significant detriment to their quality of life. For example, visual hallucinations have been reported to cause problems with sleep, diet, education and work.^11^ The purpose of this review is to describe aspects of the psychological and social impact of CBS, and summarise factors influencing the psychological and social wellbeing of people living with CBS.

## Psychological aspects of hallucinations

There is wide variability in patients’ emotional response to hallucinations. Some individuals describe being overcome by a crippling sense of fear during hallucinatory episodes; however, it is not uncommon for individuals to report pleasant, enjoyable reactions during hallucinations.^1,17^ A large-scale questionnaire study (*n* = 360) found that over 70% of patients may feel impartial and neither fear nor enjoy their hallucinations, while approximately one-quarter describe their hallucinations as unpleasant.^18^ Similarly, Cox and ffytche found that 32% of patients (*n* = 156/492) were negatively affected by their hallucinations, which was associated with more frequent and longer-lasting episodes.^16^ Patients are more likely to feel concerned when hallucinations are of a frightening and disturbing nature.^17^ In addition, unwelcome and intrusive hallucinations cause patients to feel anxious. For example, CBS phenomenology may include prosopometamorphopsia whereby facial features become grossly distorted, or feelings of claustrophobia due to trapping imagery.^19^ Such complex hallucinations can occasionally present in tandem with a difficulty tolerating symptoms, and may be associated with chronic depressive symptoms.^20^

Studies consistently indicate that around one-third of patients report experiencing negative outcomes such as distress and fear, especially throughout initial onset of symptoms.^4,5,16,21^ Patients experiencing hallucinations are statistically significantly more likely to report feelings of depression compared to those without hallucinations.^22^ Yet, similar comparison studies have found no relationship between CBS and depression.^23^ These discrepancies highlight some of the methodological challenges when assessing the psychological impact of CBS. For example, depressive symptoms may already be highly prevalent among the visually impaired;^24^ thus, careful disentanglement of depression caused truly by CBS, and underlying depression linked to co-morbidities is required in order to understand the true impact of the condition. In addition, evidence suggests there may be distinct periods where ophthalmic patients are more likely to display negative emotions, such as around the time of diagnosis and towards later end-stage disease.^25,26^ Around one-quarter of CBS patients report feelings of anxiety in response to hallucinations.^1^ Anxiety can be characterised as excessive worry and feelings of tension, often associated with somatic symptoms such as restlessness, difficulties with concentration, and sleep disturbances. This association is unsurprising given the sometimes-intrusive nature of hallucinations in CBS. Patients experiencing visual hallucinations have been found to score poorly in comparison to visually impaired controls on the General Health Questionnaire (GHQ), a measure of anxiety, depression and social dysfunction.^27^ These patients returned poor scores on the GHQ even when visual acuity was mostly preserved, suggesting emotional distress in the CBS group was not due to more advanced visual impairment. A retrospective study of 31 people with CBS found that the highest levels of anxiety were among those being treated with anxiety medications such as benzodiazepines and selective serotonin reuptake inhibitors.^20^

Poor psychological outcomes, such as anxiety and loneliness, are a recurrent feature in studies on the impact of CBS.^1,19^ However, it is equally important to recognise that a significant proportion of patients report being unperturbed by their hallucinations. One explanation for this could be habituation to CBS over time, as evidence suggests patients with long-standing CBS do not report greater negative outcomes.^16^ This is perhaps due to patients with long-term or refractory CBS being better equipped with coping strategies or possessing greater resilience to hallucinations. It is likely that the psychological repercussions of CBS are dependent on a variety of factors, including but not limited to the frequency and length of hallucination episodes, the intrusive and bothersome nature of hallucinations, disturbing characteristics of the hallucinations and the misattribution of hallucinations to neurological conditions. Greater understanding as to why some patients exhibit poor psychological adjustment to hallucinations while others remain untroubled will help improve clinical services by identifying individuals that may benefit from further intervention.

## Hallucination phenomenology

**Figure 2.**
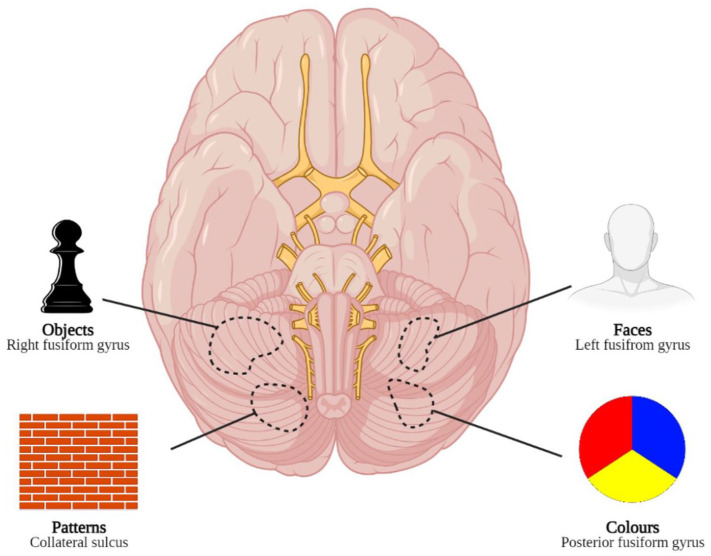
A ventral view of the brain together with types of hallucinations associated with the different areas of specialisation. Lines are used to aid anatomical localisation. Adapted from ffytche and colleagues^31^

Hallucinations in CBS are not static; observance of simple geometric patterns may progress to become more visually complex stimuli which may become persistent and disruptive to quality of life. A number of theories have emerged regarding the casual factors for onset of complex hallucinations. For example, analysis of the neural basis of CBS suggests a cascade effect, whereby more extensive visual loss is associated with more complex and enduring hallucinations.^28^ Yet, several studies have concluded that poor visual acuity is not the primary risk factor for CBS, and complex hallucinations can occur among patients with largely preserved vision.^18,29,30^ Other theories suggest the content of visual hallucinations reflects the functional specialisation of the activated region of the brain. For example, hallucinations containing colour are associated with activity in the posterior fusiform gyrus, whereas black-and-white hallucinations are associated with activity behind and above this region. Textures such as brickwork, fencing and map-like terrain are associated with activity around the collateral sulcus. Hallucinations of faces and objects are associated with activity in the left middle fusiform gyrus and right middle fusiform gyrus, respectively^31^ (Figure 2).

In addition to these pathophysiological processes, there is evidence to indicate external factors such as stressful life events and personal circumstances may catalyse onset of complex visual hallucinations. For example, a case study describes how a patient’s previously benign and short-lasting hallucinations became atypical following an evacuation during the Black Saturday bushfires disaster which led to 173 fatalities in southern Australia.^32^ Specifically, the patient’s typical hallucinations of intricate blue designs upon white backgrounds were replaced with vivid and gruesome facial hallucinations.^33^ Environmentally triggered changes in hallucination stimuli may implicate acute or post-traumatic stress in the development of complex hallucinations. This argument is supported by recent survey findings which identified repeated exposure to concerning media coverage during the coronavirus pandemic as a potential trigger for distressing changes in disease phenomenology in patients with CBS.^19^

## Awareness of CBS

Despite the high prevalence of CBS, awareness among physicians remains low. A cross-sectional survey of 499 family physicians in Canada revealed that 55% were not at all aware of CBS and that 85% never discussed the possibility of CBS with patients presenting with low vision.^34^ These numbers are not encouraging. Learning that visual hallucinations are not indicative of mental illness often ignites hope for patients with CBS, offering much needed reassurance regarding their individual health status.^1,35,36^ In addition, accurate diagnosis is critical to ensure optimal assessment of patient needs and appropriately tailored management strategies.^37,38^ A chronic absence of clinician awareness is also highlighted in patients’ reflections on their medical consultations. For example, among those who present to clinicians for medical advice, one-third of patients believed the professional was unsure or did not know about CBS.^16^ This is significant because receiving high quality, accurate, and consistent information from healthcare professionals about hallucinations in CBS is purported to reduce the associated negative sequalae.^16^ As such, presenting to a team of well-informed healthcare professionals may make the difference between patients successfully adjusting to their hallucinations with a high degree of resilience, or being poorly equipped to self-manage the condition after receiving inferior or inaccurate information. Patients may present to a range of medical and allied healthcare professionals including consultant ophthalmologists, fellows, or optometrists; hence, education is required for all those interacting with patients in high-risk groups within the hospital eye service and in the community, including the primary care setting and social services.

Awareness of CBS among patients with visual impairment is also low. A study of patients attending a Danish retina clinic found most were largely unfamiliar with CBS (24 of a total 200 patients; 12%). Strikingly, of 176 patients who were unfamiliar with CBS, 13.1% were found to have a history of CBS symptoms.^39^ The authors identified that being a patient at a low vision clinic and being educated to university level were independently associated with higher CBS awareness. Extent of internet usage was not associated with greater awareness of CBS, which the authors describe as an unexpected finding given the large amount of information on CBS available online. This finding could be explained by issues with the accessibility of online resources, suggesting the quality of patient-facing materials may be significantly more meaningful than the quantity of resources. Best-corrected visual acuity is also associated with an increased awareness of CBS, independent of whether or not the patient attends a low vision clinic.^39^ This relationship is supported by the low awareness of CBS among patients with conditions where patterns of visual field loss typically affect peripheral vision and thus visual acuity is preserved, such as glaucoma.^12^

A survey of 492 people with CBS found that 67% had not heard of the condition at symptom onset.^16^ This is particularly concerning as only 50% of patients attributed their hallucinations to sight loss, while the remainder attributed visual hallucinations to disorders other than sight loss, including mental illness and Alzheimer’s disease. Patients without prior knowledge of the condition often attribute their symptoms to other causes, including a perceived decline in cognitive functioning, or a psychiatric condition. For example, one-third of patients report being fearful of impending ‘insanity’ due to their hallucinations, while 63% feared being labelled ‘insane’ if they were to disclose their hallucinations.^17^ Patients’ attribution of hallucinations to mental illness is associated with negative impact on wellbeing and may also render patients reluctant to discuss their symptoms further.^16^ It is rare for patients who are unaware of CBS to voluntarily divulge their hallucinatory experiences;^17^ this underreporting potentially impacts upon clinician knowledge and awareness of CBS. For example, clinicians with the greatest self-reported awareness of CBS typically have more frequent contact with this patient cohort.^34^

Variability in clinician and patient awareness of CBS illuminates the vital role of education going forward. Education and reassurance play key roles in improving patient quality of life,^13,16^ but are dependent on clinician knowledge, necessitating enhanced clinician training. Targeted training for medical professionals may have limited reach due to the low rate of advice-seeking among CBS patients.^16^ However, it is likely that patients’ reluctance to share their symptoms with medical professionals stems from their own lack of awareness, coupled with a stigma surrounding visual hallucinations. Directly questioning and advising all visually impaired patients about the potential occurrence of hallucinations may counter patients’ reluctance to initiate advice-seeking behaviour and enable them to manage hallucinations more effectively.^17^ A recent randomised controlled trial carried out in Austria shows that patients benefit from clinician advice and explanation about CBS, and (with a small sample, *N* = 34) found psychiatric evaluation to be no more helpful than advice provided by an ophthalmologist.^40^ Other beneficial strategies include signposting patients to support groups. One study found that the vast majority of members of the Macular Society (87%), a charity support group for people with macular disease, were knowledgeable of CBS,^16^ compared to only 29% of a wider clinical population of patients with age-related macular degeneration.^41^ Patients attending low vision clinics are also more likely to be familiar with the condition,^39^ suggesting that referrals to such clinics and organisations improve patient awareness.

## New developments in measuring and managing the impacts of CBS

**Figure 3.**
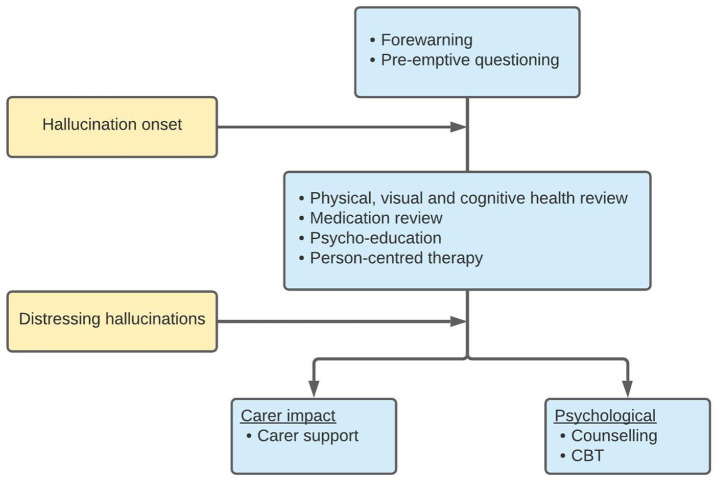
The staged approach to managing visual hallucinations in Charles Bonnet syndrome (CBS). Adapted from the SHAPED consensus framework.^42^ Yellow boxes indicate hallucination characteristics and blue the therapeutic targets. Cognitive behavioural therapy (CBT).

The current expanding body of literature on CBS is promising. Raising the scientific profile of CBS creates opportunity to increase knowledge and understanding of the condition, which ultimately has potential to translate into enhanced patient care. Two recent developments in the field are particularly notable. First, the design of a consensus framework for the management of visual hallucinations in high burden areas, including CBS^42^ (Figure 3). The framework was developed following the SHAPED trial (Study of Hallucinations in Parkinson’s disease, Eye disease and Dementia) and suggests management should begin prior to the onset of hallucinations by informing patients of their susceptibility, and pre-emptive questioning to encourage reporting upon symptom manifestation. This ‘forewarned is forearmed’ approach sets a precedent of open dialogue surrounding hallucinations and raising awareness among susceptible populations which will help dismantle barriers and reduce hesitancy around discussing hallucinations. The framework goes on to provide a staged approach to patient management (see below).

A further noteworthy advancement in CBS is the development of the French-language *Questionnaire de repérage du syndrome de Charles Bonnet* (QR-SCB; in English, Charles Bonnet Syndrome Screening Questionnaire).^43^ The 55-item instrument was developed to facilitate identification of CBS and to identify the patient’s need for intervention. Initial validation suggests the instrument has respectable psychometric properties when used alongside clinical judgement (sensitivity 1.00; specificity 0.77). The QR-SCB is organised in eight dimensions including: screening; characteristics of hallucinations; psychological impact; psychopathological origin; coping strategies; context of appearance of hallucinations; time-related matters; psychological support. The tool could be used to identify CBS and describe various dimensions of the patient experience. Administration time is lengthy compared to other outcomes measures used in ophthalmology, and for the purposes of screening, the tool may be most beneficial as an adjunct to clinical assessments. Additional research efforts are required to better understand the psychometric properties of the QR-SCB, as well as appropriate validation of any emerging translated versions of the tool. Nevertheless, the QR-SCB could be a clinically useful tool in CBS patient care pathways and intervention planning, and its development illustrates an encouraging step towards elevating awareness and understanding of CBS.

## Social isolation

Social isolation refers to a person’s separation from significant others, groups, activities and situations. A combination of attributes can be used to measure social isolation including number of social contacts, feelings of belonging, fulfilling relationships, engagement with others, and the quality of members within social networks.^44^ Involuntary social isolation can be an alienating and lonely experience with negative consequences for health. In the context of CBS, theories suggest sensory deprivation as a result of social isolation may be partly responsible for visual hallucinations. For example, several studies associate living alone with onset of visual hallucinations,^29,45,46^ however these findings are not always replicated.^18,41^ One study suggests that loneliness in the sense of poor quality of social relationships – rather than simply the number of social contacts may be associated with CBS. When considering theories regarding the aetiology of CBS, for instance that hallucinations occur as a response to changing sensory input to specific areas of the brain,^15,47,48^ it makes sense clinically that social isolation may have implications for CBS. While it is difficult to elucidate the precise role social isolation plays in the genesis of CBS, there is convincing evidence to indicate an association. For example, temporary cessation of hallucinations during periods of hospitalisation have been attributed to breaks from isolation.^1^ Furthermore, isolation and loneliness during the COVID-19 pandemic were associated with exacerbations in disease phenomenology for people with CBS.^19^

## Management

An excellent overview of current clinical practice for CBS management was provided by Carpenter and colleagues,^13^ and we briefly summarise the therapeutic approaches here. Forewarning people with vision loss that they may encounter hallucinations can be an effective proactive management strategy in CBS. The SHAPED consensus framework (Figure 3) proposes that management ought to begin before symptom onset with forewarning, education and questioning to encourage reporting.^42^ Awareness of CBS at the time of onset offers psychological protection and is predicted to reduce negative outcomes by 20%.^16^ As such, raising awareness and delivering educational campaigns, such as through patient support groups (www.charlesbonnetsyndrome.uk/), are meaningful pre-emptive strategies to bolster psychological readiness for patients experiencing hallucinations. Patient support groups also facilitate networking and sharing of experiences and useful resources, such as behavioural exercises which may help to reduce CBS symptoms. For example, such exercises include frequent blinking or rapid eye movements, changing light levels to increase visual input, and alerting/distraction techniques.^49^

The SHAPED framework incorporates further intervention plans for individuals with particularly distressing hallucinations or refractive cases, including counselling, carer support and consideration of pharmacological treatment.^42^ A number of medical therapeutic candidates have been associated with reducing or eliminating visual hallucinations in CBS, including antipsychotics, anticonvulsants, anti-anxiety, and selective serotonin reuptake inhibitors.^50–52^ There are no definitive medication recommendations in CBS; hence they are not included in the SHAPED framework. Application of low frequency repetitive transcranial magnetic stimulation to the occipital lobe in a single case was apparently effective at supressing hallucinations.^53^ However, this technique has not yet entered clinical trials and therefore lacks robust evidence-base for efficacy.

Optimisation of vision may help prevent and resolve CBS. Small-scale studies report cessation of visual hallucinations following cataract removal and laser treatment,^51,54^ although the extent to which restoration of vision reduces the incidence of CBS is difficult to assess directly. It is, however, gratifying that the mainstay when treating patients with visual impairment is to improve vision where possible and prevent further deterioration, potentially minimising the risk of CBS. However, recent data highlighting the rationing of cataract procedures in the United Kingdom is concerning,^55^ given that restricted access to the procedure will have possible repercussions for the epidemiology of CBS.

## Conclusion

The prevalence of CBS may be higher, and the content of hallucinations more disturbing for patients, than originally thought. Nonetheless, awareness of the condition remains low, including among healthcare professionals and low-vision patients. Findings from this review indicate that lack of awareness of CBS may exacerbate patients’ anxiety and has a detrimental effect on daily living and wellbeing. The stigma associated with visual hallucinations might adversely impact advice-seeking and symptom sharing by CBS patients and might exacerbate isolation and feelings of loneliness, themselves risk factors for CBS. A staged approach to management is recommended, to begin education and reassurance before the potential onset of CBS symptoms. This review highlights the need to prioritise education and training of clinicians in order to facilitate a pre-emptive approach to CBS management.
